# Physician Peer Influence on Salpingectomy Uptake for Tubal Sterilization and Ovarian Cancer Prevention

**DOI:** 10.1001/jamanetworkopen.2025.32998

**Published:** 2025-09-22

**Authors:** Xiao Xu, Jessica B. Long, Craig Evan Pollack, Vrunda B. Desai, Cary P. Gross, Erica S. Spatz, Jason D. Wright

**Affiliations:** 1 Department of Obstetrics and Gynecology, Columbia University Vagelos College of Physicians and Surgeons, New York, New York; 2 Herbert Irving Comprehensive Cancer Center, Columbia University, New York, New York; 3 Department of Internal Medicine, Yale School of Medicine, New Haven, Connecticut; 4 Department of Health Policy and Management, Johns Hopkins Bloomberg School of Public Health, Baltimore, Maryland; 5 Johns Hopkins School of Nursing, Baltimore, Maryland; 6 CooperSurgical, Inc, Trumbull, Connecticut; 7 Department of Obstetrics, Gynecology and Reproductive Sciences, Yale School of Medicine, New Haven, Connecticut

## Abstract

**Importance:**

Prophylactic removal of the fallopian tubes (opportunistic salpingectomy) in lieu of conventional methods of tubal sterilization helps reduce ovarian cancer risk.

**Objective:**

To examine how peer influence among physicians is associated with opportunistic salpingectomy uptake at the time of postpartum and interval sterilizations.

**Design, Setting, and Participants:**

This retrospective cohort study used insurance claims data from the Blue Cross Blue Shield Axis database. Participants were patients aged 18 to 49 years who underwent a tubal sterilization after childbirth (postpartum sterilization) or unrelated to pregnancy (interval sterilization) from January 2020 to December 2022 and whose operating surgeon did not perform opportunistic salpingectomy at baseline (2017-2019).

**Exposures:**

Applying a Louvain clustering methodology, physician patient-sharing networks were constructed based on their billing of the same patients in claims data at baseline (2017-2019). For surgeons who did not use opportunistic salpingectomy at baseline, the rate of opportunistic salpingectomy use among peer physicians in the same network at baseline was measured.

**Main Outcomes and Measures:**

Opportunistic salpingectomy was defined as removal of both (or the remaining side of) fallopian tubes with preservation of the ovaries.

**Results:**

The overall sample included 4520 patients undergoing postpartum sterilization and 3376 patients undergoing interval sterilization in 2020 to 2022, with 4173 (92.3%) of the postpartum sterilization sample involving a cesarean delivery. Most patients in these 2 samples (3520 patients [77.9%] and 2599 patients [77.0%], respectively) were aged 30 to 49 years. In the postpartum sterilization sample, having peer physicians in the highest (vs lowest) quartile of baseline opportunistic salpingectomy rate was associated with higher odds of receiving opportunistic salpingectomy for postpartum sterilization (13.6% vs 5.6%; adjusted odds ratio [aOR], 2.17; 95% CI, 1.20-3.92; *P* < .001). In the interval sterilization sample, having peer physicians in the highest (vs lowest) quartile of baseline opportunistic salpingectomy rate was also associated with higher odds of receiving opportunistic salpingectomy for interval sterilization (42.3% vs 19.3%; aOR, 4.16; 95% CI, 1.98-8.77; *P* < .001).

**Conclusions and Relevance:**

In this retrospective cohort study of postpartum and interval sterilizations, surgeons who previously shared patients with other physicians with high rates of opportunistic salpingectomy use were more likely to adopt opportunistic salpingectomy in their own subsequent practice. These results suggest physician peer influence in salpingectomy uptake.

## Key Points

**Question:**

Is peer influence among physicians associated with their adoption of opportunistic salpingectomy as a method of tubal sterilization to help reduce ovarian cancer risk?

**Findings:**

In this cohort study, among 4520 patients undergoing postpartum sterilization and 3376 patients undergoing interval sterilization, there were 2.17-fold and 4.16-fold significantly higher odds of receiving opportunistic salpingectomy, respectively, if operating surgeons previously shared patients with other physicians who had the highest quartile of baseline opportunistic salpingectomy rate.

**Meaning:**

These findings suggest physician peer influence may play an important role in salpingectomy uptake for ovarian cancer prevention.

## Teaser

This cohort study evaluates how peer influence among physicians is associated with opportunistic salpingectomy in postpartum and interval sterilizations.

## Introduction

Ovarian cancer causes more deaths than most other gynecologic cancer.^1^ As there is no routine screening, primary prevention is particularly beneficial. Evidence suggests that high grade serous ovarian carcinoma (the most common and aggressive type of ovarian cancer) originates from the fallopian tubes rather than the ovaries.^2,3^ Therefore, for women after completing childbearing, opportunistic salpingectomy (OS)—removing both fallopian tubes (in lieu of the conventional method of sterilization via tubal interruption)—offers dual benefits of sterilization and ovarian cancer risk reduction.^2,3^ With more than 600 000 tubal sterilizations in the US each year,^4^ OS presents great potential for cancer prevention.

In 2015, the American College of Obstetricians and Gynecologists recommended counseling patients about OS as a means of sterilization.^2,3^ Since then, use of OS increased rapidly; OS at the time of cesarean delivery rose from 4.6% in the last quarter of 2015 to 13.2% in the last quarter of 2018 nationwide.^5^ Yet clinicians varied widely in OS uptake^6,7,8^ with physicians’ differential beliefs about OS for ovarian cancer risk reduction, complication risk, and surgical time being major contributing factors.^6,7,9,10,11,12,13^ Given varying knowledge gaps among physicians about OS,^14^ there is likely substantial subjectivity in their beliefs prone to peer influence.^15,16^ Consistent with prior literature on social contagion among physicians in their adoption of new therapies and evidence-based medicine,^17,18,19,20^ we hypothesized there is peer influence among physicians in their adoption of OS as a means of permanent sterilization.

## Methods

### Overview

This was a retrospective cohort study using deidentified commercial claims data across the US from the Blue Cross Blue Shield (BCBS) Axis database. We captured physician peer relationship by constructing physician patient-sharing networks (ie, informal professional networks of physicians who frequently shared patients with each other regardless of their organizational affiliation or payer contract).^21,22^ Physicians practicing in the same network were considered peers. Focusing on surgeons who did not perform OS for tubal sterilization in 2017 to 2019 (time [T] 1), we examined whether there was an association between their peer physicians’ OS practice in T1 and their own subsequent use of OS in 2020 to 2022 (T2). This study was deemed exempt from review by the Yale University institutional review board with a waiver of informed consent because it was a secondary analysis of deidentified data and was approved by Columbia University institutional review board as well. The conduct of this study followed the Strengthening the Reporting of Observational Studies in Epidemiology (STROBE) reporting guideline.

### Data and Sample

The BCBS Axis database contains integrated data from the BCBS system across the US. It provides comprehensive information on each enrollee’s sociodemographic characteristics and medical care. We identified women aged 18 to 49 years who underwent tubal sterilization from 2020 to 2022 and had continuous insurance coverage in the previous 12 months (T2 sterilization sample) (eFigure in Supplement 1). This included postpartum sterilization (ie, sterilization performed at a childbirth hospitalization) and interval sterilization (ie, sterilization performed in an outpatient setting unrelated to pregnancy). Tubal sterilization was determined using procedure codes for tubal ligation and salpingectomy in conjunction with a diagnosis code for encounter of sterilization (without diagnosis of abortion or ectopic pregnancy) (relevant codes in eTable in Supplement 1). To study OS adoption, we limited the T2 sterilization sample to patients whose operating surgeon were baseline nonusers (ie, did not perform OS in T1).

Using diagnosis and procedure codes in the previous 12 months and the sterilization encounter, we excluded patients who had (1) a hysterectomy, history of tubal ligation, or acquired absence of genital organs; (2) ovarian and/or fallopian tube disorders (who might undergo salpingectomy and/or oophorectomy for therapeutic reasons); or (3) cancer or elevated risk for cancer (to focus on women at low to average risk for ovarian cancer who would benefit the most from OS). We also excluded women who had a salpingectomy and/or oophorectomy in the previous 12 months or whose surgeon had unknown identity or implausible or unknown specialty (eg, anesthesiology or pathology).

Using data from 2017 to 2019, we also constructed a broad sample of women aged 18 to 64 years with continuous insurance coverage in the 12 months before the index date to depict the physician-peer relationship (T1 patient-sharing sample). The index date was the date of sterilization (if patient underwent tubal sterilization) or a randomly assigned date otherwise. A subset of these patients who underwent sterilization from 2017 to 2019, were aged 18 to 49 years, and had continuous insurance coverage in the previous 12 months formed the T1 sterilization sample, which was used to calculate peer physicians’ baseline OS rate. These patients met the same other eligibility criteria as described for the T2 sterilization sample.

### Physician Patient-Sharing Network

**Figure 1.**
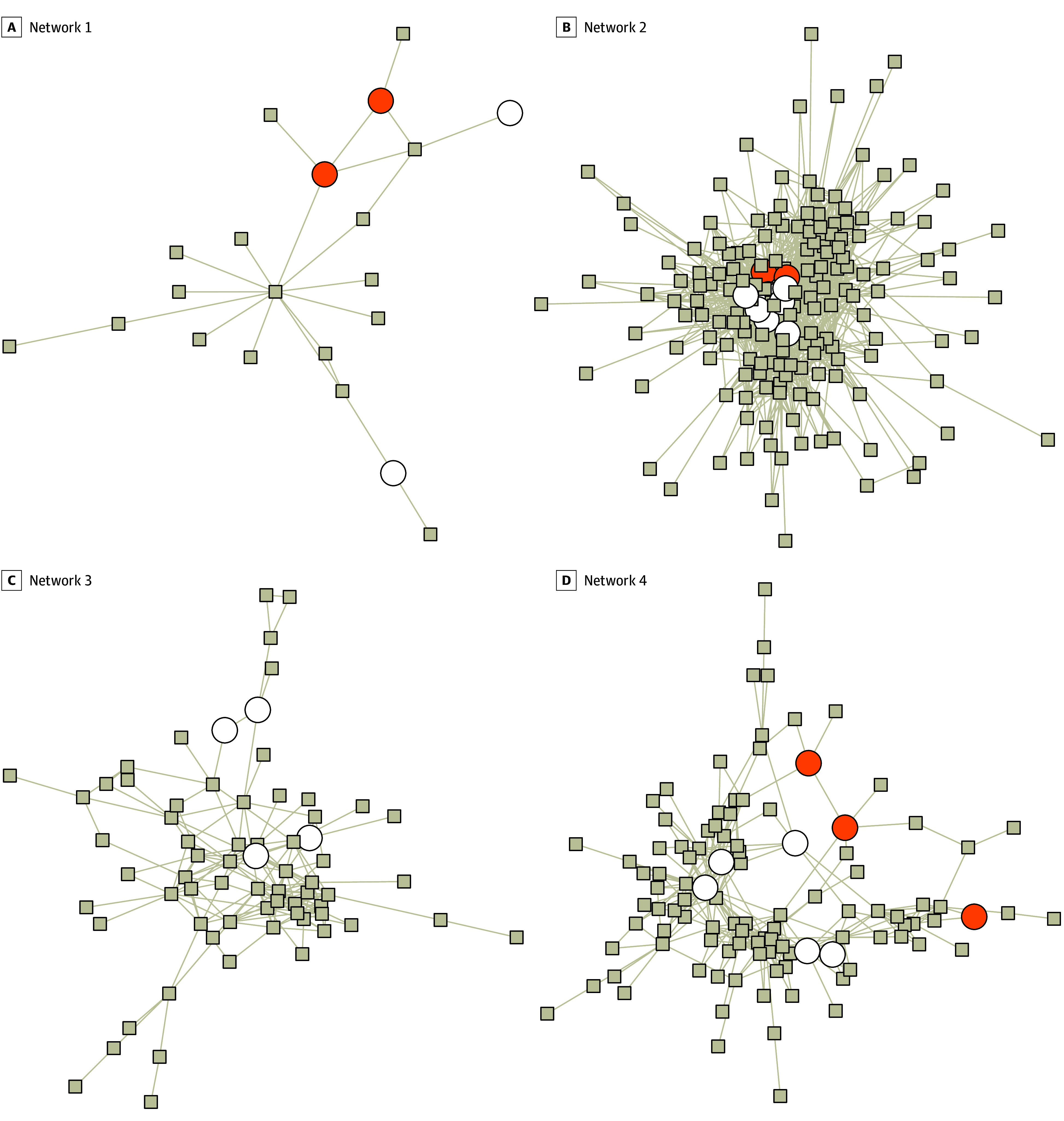
Illustration of Physician Patient-Sharing Networks in 1 Example Hospital Referral Region (HRR) at Baseline (2017-2019) This example HRR contained 4 physician patient-sharing networks that involved at least 2 physicians who performed tubal sterilization from 2017 to 2019 (time [T] 1). Circles denote physicians who performed tubal sterilization in T1, with orange circles reflecting those who used opportunistic salpingectomy (OS) in T1 and empty circles reflecting those who did not use OS in T1. Squares denote other physicians connected in the same network who did not perform tubal sterilization in T1. Lines linking the circles and squares indicate connections among physicians through patient sharing. Not shown in this figure were other physician patient-sharing networks in this HRR that did not have any physician who performed sterilization in T1 or had only 1 physician who performed sterilization in T1 (where baseline peer physician OS rate could not be calculated). A, Network 1 included 2 sterilization physicians who used OS in T1, 2 sterilization physicians who did not use OS in T1, and 17 other physicians who did not perform sterilization in T1. B, Network 2 included 2 sterilization physicians who used OS in T1, 8 sterilization physicians who did not use OS in T1, and 162 other physicians who did not perform sterilization in T1. C, Network 3 included 4 sterilization physicians who did not use OS in T1, no sterilization physician who used OS in T1, and 65 other physicians who did not perform sterilization in T1. D, Network 4 included 3 sterilization physicians who used OS in T1, 5 sterilization physicians who did not use OS in T1, and 99 other physicians who did not perform sterilization in T1.

We identified physician patient-sharing networks using the T1 patient-sharing sample (see example networks in Figures 1A-1D). Physicians who billed for the same patient on insurance claims were considered to share the patient. Following prior research and to more likely capture meaningful patient-sharing relationships,^21^ we required each physician to bill for 4 or more patients and 2 physicians to share 2 or more patients in order be considered connected. As some patients had an exceedingly large number of physicians, we capped their connections to the top 8 physicians in terms of interaction-days (corresponding to the 90th percentile) to focus on those with a clinically meaningful relationship with the patient. We included insurance claims billed by obstetricians and gynecologists, as well as urologists and primary care physicians (internal medicine, general practice, and family practice). Inclusion of the latter specialties helped enhance robustness of the networks identified while focusing on the most relevant connections. Based on physician connections through these claims, we applied a Louvain clustering methodology in the igraph package using R version 4.2.2 (R Project for Statistical Computing) to identify physician networks (stratified by hospital referral regions).^23,24^ The Louvain algorithm partitioned physicians into nonoverlapping networks by optimizing modularity (ie, the density of physician connections within networks relative to density of physician connections between networks).^23,24^ Although the nonoverlapping assumption might overlook possible interactions among some physicians between networks below our selected thresholds (eg, shared <2 patients between 2 physicians or had connections through physicians with other specialties), the networks we identified should capture the strongest connections among physicians that were most relevant for our study.

### Outcome

For each patient in the T2 sterilization sample, we measured whether they received OS as a method of sterilization (yes or no), defined as removal of the fallopian tubes with preservation of the ovaries. Since all postpartum sterilizations were inpatient procedures and had *International Classification of Diseases, Tenth Revision (ICD-10)* procedure codes available, we measured OS based on *ICD-10* procedure codes for complete salpingectomy without concurrent oophorectomy (eTable in Supplement 1). In contrast, interval sterilizations were outpatient procedures and only had *Current Procedural Terminology* (*CPT*)/Healthcare Common Procedure Coding System (HCPCS) codes available, which could not differentiate complete vs partial salpingectomy. Therefore, for interval sterilization, we measured OS based on *CPT*/HCPCS procedure codes for any salpingectomy without concurrent oophorectomy (eTable in Supplement 1). Since all patients in the T2 sterilization sample had a diagnosis code for sterilization encounter, we accepted both unilateral and bilateral salpingectomy assuming unilateral salpingectomy was for the remaining tube.

### Exposure

For each physician in the T1 sterilization sample, we calculated their own OS rate as the proportion of sterilization procedures they performed in T1 that used OS. Other physicians in T1 who were in the same network and also performed sterilizations were referred to as their peer physicians. For a given physician, we calculated the mean OS rate among their peer physicians in T1 and categorized it into quartiles, reflecting whether they shared patients with peer physicians who had low, medium low, medium high, or high OS rates at baseline. By linking physicians in the T1 and T2 sterilization samples, we measured T1 peer physicians’ OS rate for each T2 operating surgeon. This was the exposure variable of our analysis. To ensure peer physicians had a reasonable volume for calculating OS rate, we required each peer physician to perform 5 or more eligible sterilizations in T1 (eligible sterilization defined in the same way as in the T2 sterilization sample).

### Covariates

For each patient in the T2 sterilization sample, we measured their age, body mass index (BMI; calculated as weight in kilograms divided by height in meters squared), smoking status (yes or no), Elixhauser comorbidities (0, 1, or ≥2),^25,26^ census region, zip code-based social deprivation index (SDI),^27^ location in metropolitan area (yes or no), surgeon’s surgical volume (number of postpartum sterilizations and interval sterilizations, respectively, performed in T2), and year of sterilization. BMI, smoking status, and comorbidities were measured using diagnosis codes in the 12 months before and through the sterilization encounter (eTable in Supplement 1). For postpartum sterilizations, we also measured mode of delivery (cesarean vs vaginal) using procedure codes at the sterilization encounter. Selection of these covariates were consistent with prior research suggesting that patient age, socioeconomic status, geographic region, rurality of residence location, comorbidities, year, and mode of delivery (for postpartum sterilization) were associated with the use of OS.^5,28^

### Statistical Analysis

We analyzed patients in the T2 sterilization sample whose operating surgeons were baseline nonusers. Postpartum and interval sterilizations were analyzed separately because their influencing factors differed. Characteristics of patients who underwent OS and those who did not undergo OS were compared using χ^2^ tests and Wilcoxon rank sum tests. We also summarized the characteristics of physician networks identified, including number of physicians (overall and those who performed eligible sterilizations), number of eligible sterilizations, and network density (actual number of connections among its physicians divided by the maximum possible number of connections among its physicians; value range: 0 to 1, with higher values indicating more interconnected physicians).^29^

Patient-level multivariable regressions (generalized linear model with logit link and binomial distribution) were used to analyze the association between peer physicians’ OS rate in T1 (exposure variable) and patients’ likelihood of receiving OS in T2 (outcome variable). Covariates with *P* < .2 in univariate analysis were included in the regression models. Clustering of patients within surgeons and clustering of surgeons within physician networks were accounted for using nested random effects. We also tested adding the network density variable and its interaction term with peer physicians’ baseline OS rate (in quartiles) to inform whether physician peer influence was stronger in more interconnected networks.

Following prior research,^30^ we also fit null models with nested random effects (without adjustment for any covariates) to estimate patient-, surgeon-, and network-level variance. This variance decomposition informed what proportion of overall variance in OS use in T2 was explained at the patient-, surgeon-, and network-level, respectively.

Data management was conducted in SQL Server Management System version 17.0 (Microsoft). Analyses were conducted using R version 4.2.2 and Stata version 16 (StataCorp). Two-sided *P* values less than .05 were considered statistically significant.

## Results

The T2 (2020-2022) postpartum sterilization sample included 4520 eligible patients. Among them, 4173 (92.3%) had cesarean deliveries (Table 1). Most patients in these 2 samples (3520 patients [77.9%] and 2599 patients [77.0%], respectively) were aged 30 to 49 years. These patients were operated on by 1312 surgeons who did not use OS at baseline. Meanwhile, the T2 (2020-2022) interval sterilization sample included 3376 patients (Table 2) operated on by 1158 surgeons who did not use OS at baseline.

**Table 1.** Characteristics of Patients in T2 Postpartum Sterilization Sample

Characteristic	Patients, No. (%)^a^			*P* value^b^
	Overall (N = 4520)	Opportunistic salpingectomy in T2		
		Yes (n = 353)	No (n = 4167)	
Quartile of peer physicians’ rate of opportunistic salpingectomy in T1				
1 (0%-2.4%)	1097 (24.3)	61 (17.3)	1036 (24.9)	<.001
2 (>2.4%-7.8%)	952 (21.1)	41 (11.6)	911 (21.9)	
3 (>7.8%-16.9%)	1269 (28.1)	87 (24.6)	1182 (28.4)	
4 (>16.9%)	1202 (26.6)	164 (46.5)	1038 (24.9)	
Age, y				
18-29	1000 (22.1)	50 (14.2)	950 (22.8)	.001
30-34	1661 (36.7)	129 (36.5)	1532 (36.8)	
35-39	1495 (33.1)	142 (40.2)	1353 (32.5)	
40-49	364 (8.1)	32 (9.1)	332 (8.0)	
Mode of delivery				
Vaginal	347 (7.7)	10 (2.8)	337 (8.1)	<.001
Cesarean	4173 (92.3)	343 (97.2)	3830 (91.9)	
Body mass index^c^				
<25	2792 (61.8)	221 (62.6)	2571 (61.7)	.13
25-29	236 (5.2)	27 (7.6)	209 (5.0)	
30-39	843 (18.7)	60 (17.0)	783 (18.8)	
≥40	649 (14.4)	45 (12.7)	604 (14.5)	
Smoking status				
No	3946 (87.3)	294 (83.3)	3652 (87.6)	.02
Yes	574 (12.7)	59 (16.7)	515 (12.4)	
Elixhauser category				
0	2577 (57.0)	185 (52.4)	2392 (57.4)	.16
1	1300 (28.8)	116 (32.9)	1184 (28.4)	
≥2	643 (14.2)	52 (14.7)	591 (14.2)	
Zip code in metropolitan area				
No	1342 (29.7)	94 (26.6)	1248 (29.9)	.19
Yes	3178 (70.3)	259 (73.4)	2919 (70.1)	
Census region				
Midwest	884 (19.6)	101 (28.6)	783 (18.8)	<.001
Northeast	421 (9.3)	77 (21.8)	344 (8.3)	
South	3067 (67.9)	158 (44.8)	2809 (69.8)	
West	148 (3.3)	17 (4.8)	131 (3.1)	
Social deprivation index score				
<25 (least vulnerable)	1106 (24.5)	109 (30.9)	997 (23.9)	<.001
25-50	1189 (26.3)	95 (26.9)	1094 (26.3)	
50-75	1374 (30.4)	82 (23.2)	1292 (31.0)	
>75 (most vulnerable)	764 (16.9)	50 (14.2)	714 (17.1)	
Unknown	87 (1.9)	17 (4.8)	70 (1.7)	
Operating surgeon’s postpartum sterilization volume, median (IQR)^d^	5 (3-9)	4 (3-7)	5 (3-9)	<.001
Year of sterilization				
2020	1625 (36.0)	100 (28.3)	1525 (36.6)	.01
2021	1570 (34.7)	139 (39.4)	1431 (34.3)	
2022	1325 (29.3)	114 (32.3)	1211 (29.1)	

Abbreviations: T1, time 1 (2017-2019); T2, time 2 (2020-2022).

^a^ Percentages may not add to 100% due to rounding.

^b^ *P* values were based on χ^2^ tests (for categorical variables) and Wilcoxon rank sum tests (for continuous variables).

^c^ Calculated as weight in kilograms divided by height in meters squared.

^d^ Number of eligible postpartum sterilizations performed by the operating surgeon in T2.

**Table 2.** Characteristics of Patients in Time T2 Interval Sterilization Sample

Characteristic	Patients, No. (%)^a^			*P* value^b^
	Overall (N = 3376)	Opportunistic salpingectomy in T2		
		Yes (n = 902)	No (n = 2474)	
Quartile of peer physicians’ rate of opportunistic salpingectomy in T1				
1 (0%-2.4%)	808 (23.9)	156 (17.3)	652 (26.4)	<.001
2 (>2.4%-7.8%)	865 (25.6)	144 (16.0)	721 (29.1)	
3 (>7.8%-16.9%)	868 (25.7)	249 (27.6)	619 (25.0)	
4 (>16.9%)	835 (24.7)	353 (39.1)	482 (19.5)	
Age, y				
18-29	777 (23.0)	185 (20.5)	592 (23.9)	.11
30-34	921 (27.3)	248 (27.5)	673 (27.2)	
35-39	936 (27.7)	272 (30.2)	664 (26.8)	
40-49	742 (22.0)	197 (21.8)	545 (22.0)	
Body mass index^c^				
<25	1880 (55.7)	525 (58.2)	1355 (54.8)	.13
25-29	330 (9.8)	87 (9.6)	243 (9.8)	
30-39	763 (22.6)	179 (19.8)	584 (23.6)	
≥40	403 (11.9)	111 (12.3)	292 (11.8)	
Smoking status				
No	2769 (82.0)	744 (82.5)	2025 (81.9)	.67
Yes	607 (18.0)	158 (17.5)	449 (18.1)	
Elixhauser category				
0	2132 (63.2)	560 (62.1)	1572 (63.5)	.03
1	803 (23.8)	240 (26.6)	563 (22.8)	
≥2	441 (13.1)	102 (11.3)	339 (13.7)	
Zip code in metropolitan area				
No	1038 (30.7)	237 (26.3)	801 (32.4)	.001
Yes	2338 (69.3)	665 (73.7)	1673 (67.6)	
Census region				
Midwest	679 (20.1)	223 (24.7)	456 (18.4)	<.001
Northeast	326 (9.7)	173 (19.2)	153 (6.2)	
South	2239 (66.3)	447 (49.6)	1792 (72.4)	
West	132 (3.9)	59 (6.5)	73 (3.0)	
Social deprivation index score				
<25 (least vulnerable)	655 (19.4)	242 (26.8)	413 (16.7)	<.001
25-50	938 (27.8)	264 (29.3)	674 (27.2)	
50-75	1121 (33.2)	255 (28.3)	866 (35.0)	
>75 (most vulnerable)	615 (18.2)	120 (13.3)	495 (20.0)	
Unknown	47 (1.4)	21 (2.3)	26 (1.1)	
Operating surgeon’s interval sterilization volume, median (IQR)^d^	4 (2-7)	3 (2-6)	5 (3-8)	<.001
Year of sterilization				
2020	1028 (30.5)	190 (21.1)	838 (33.9)	<.001
2021	1146 (33.9)	284 (31.5)	862 (34.8)	
2022	1202 (35.6)	428 (47.5)	774 (31.3)	

Abbreviations: T1, time 1 (2017-2019); T2, time 2 (2020-2022).

^a^ Percentages may not add to 100% due to rounding.

^b^ *P* values were based on χ^2^ tests (for categorical variables) and Wilcoxon rank sum tests (for continuous variables).

^c^ Calculated as weight in kilograms divided by height in meters squared.

^d^ Number of eligible interval sterilizations performed by the operating surgeon in T2.

For these T2 operating surgeons, Table 3 summarizes the characteristics of their patient-sharing networks at baseline (2017-2019). Surgeons of the postpartum sterilization sample came from 528 patient-sharing networks. These networks had a median (IQR) of 12.0 (7.0-21.5) physicians who performed sterilization at baseline. Likewise, operating surgeons of the interval sterilization sample came from 494 patient-sharing networks (median [IQR], 12.0 [7.0-21.0] physicians who performed sterilization at baseline).

**Table 3.** Physician Patient-Sharing Networks

Measure	Post partum sterilization (n = 528 networks)	Interval sterilization (n = 494 networks)
Characteristics of physician patient-sharing networks at baseline (2017-2019), median (IQR)		
No. of physicians per network^a^	164.5 (97.0-293.5)	157.0 (92.0-284.0)
No. of physicians who performed eligible sterilizations per network	12.0 (7.0-21.5)	12.0 (7.0-21.0)
No. of eligible sterilizations per network	65.0 (38.0-115.0)	65.5 (39.0-115.0)
Density of the network^b^	0.07 (0.05-0.12)	0.08 (0.05-0.12)
Decomposition of variance in opportunistic salpingectomy use in 2020-2022, % (95% CI)		
Proportion of variance explained at patient level	43.5 (36.8-53.0)	25.0 (20.6-31.8)
Proportion of variance explained at surgeon level	34.1 (21.5-44.7)	43.3 (33.6-51.8)
Proportion of variance explained at physician patient-sharing network level	22.4 (11.9-32.2)	31.6 (21.8-40.3)

^a^ Included all physicians in the network (primary care physicians, obstetricians and gynecologists, and urologists) regardless of whether they performed sterilization procedures.

^b^ Density of a network was calculated as the actual number of connections among its physicians divided by the maximum possible number of connections among its physicians. Network density takes a value ranging from 0 to 1 with higher values indicating more interconnected physicians in the network.

Overall, 353 of the 4520 patients (7.8%) in the T2 postpartum sterilization sample and 902 of the 3376 patients (26.7%) in the T2 interval sterilization sample received OS in 2020 to 2022 (Tables 1 and 2). Variance decomposition suggested that physician networks explained 22.4% (95% CI, 11.9%-32.2%) and 31.6% (95% CI, 21.8%-40.3%) of the variation in OS use among postpartum and interval sterilizations in T2, respectively (Table 3).

We divided operating surgeons into quartiles based on empirical distribution of their peer physicians’ mean baseline OS rate (0%-2.4%, >2.4%-7.8%, >7.8%-16.9%, and >16.9%). Compared with patients in T2 whose operating surgeon had peer physicians with the lowest quartile of baseline OS rate, a larger proportion of patients in T2 whose operating surgeon had peer physicians in the highest quartile of baseline OS rate received OS (61 of 1097 [5.6%] vs 164 of 1202 [13.6%] in the postpartum sterilization sample; *P* < .001; 156 of 808 [19.3%] vs 353 of 835 [42.3%] in the interval sterilization sample; *P* < .001) (Tables 1 and 2).

**Figure 2.**
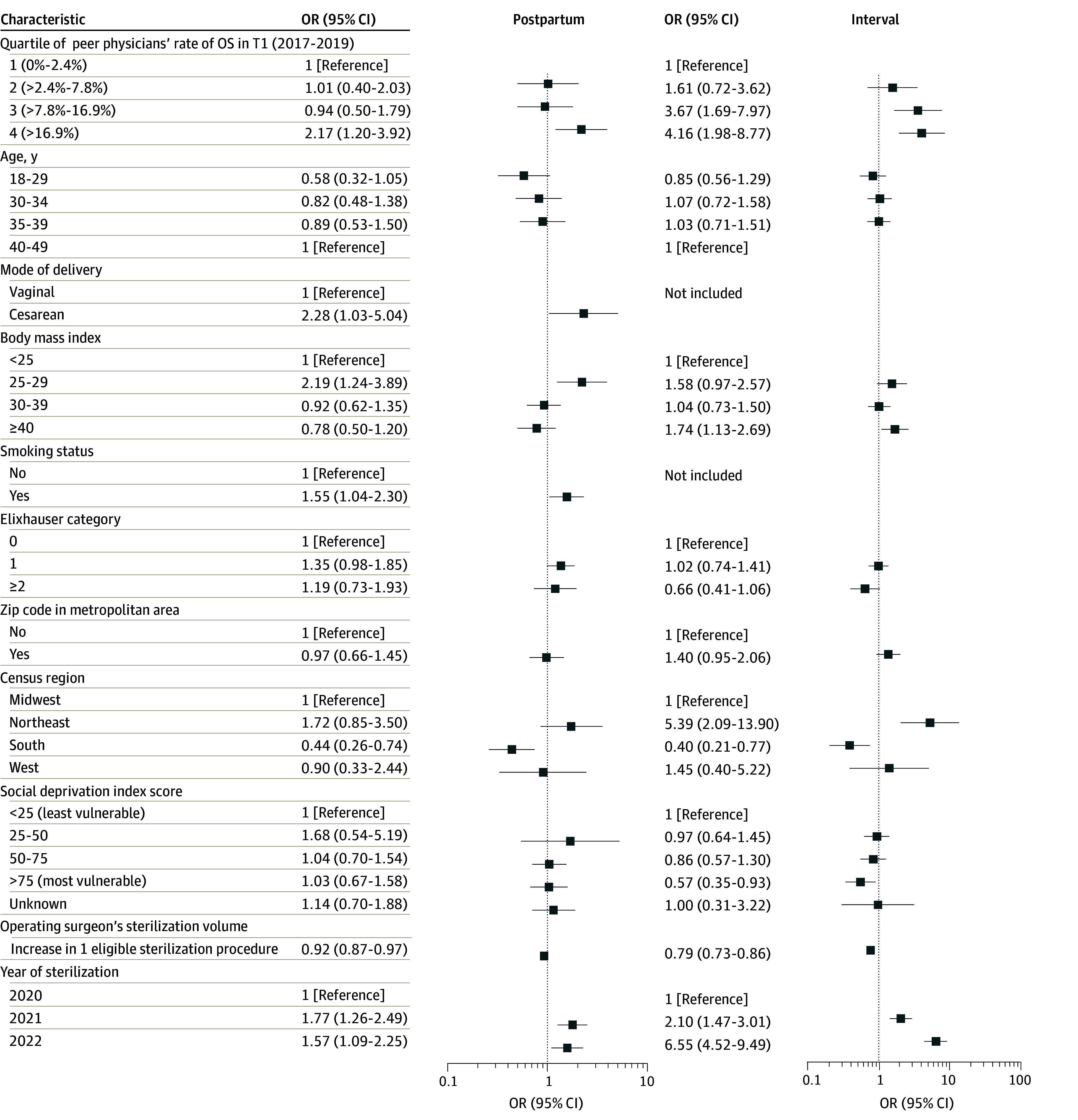
Association Between Peer Physicians’ Baseline Salpingectomy Rate and the Operating Surgeon’s Own Patients’ Likelihood of Receiving Opportunistic Salpingectomy (OS) in 2020-2022 Only covariates with *P* values less than .20 in univariate analysis were included in multivariable regression analysis. Separate regression models were estimated for patients’ likelihood of receiving OS at the time of postpartum sterilization and interval sterilization, respectively. For each regression model, all variables listed in the figure were included simultaneously. Body mass index is calculated as weight in kilograms divided by height in meters squared. OR indicates odds ratio; T, time.

These differences persisted in multivariable regression analysis adjusting for patients’ sociodemographic and clinical characteristics (Figure 2). In the T2 postpartum sterilization sample, when operated on by a surgeon whose peer physicians had the highest quartile of baseline OS rate, a patient was 2.17 (95% CI, 1.20-3.92) times more likely to receive OS in T2. In the T2 interval sterilization sample, when operated on by a surgeon whose peer physicians had the highest and second highest quartiles of baseline OS rate, a patient was 4.16 (95% CI, 1.98-8.77) and 3.67 (95% CI, 1.69-7.97) times more likely to receive OS in T2, respectively. Further evaluation of an interaction term between peer exposure and network density showed no significant interaction effect.

Multivariable regression analysis also showed other patient characteristics associated with OS use in T2. More recent years of surgery, higher BMI, Northeast location, and cesarean delivery were generally associated with higher odds of OS, whereas location in the South or areas with the most SDI vulnerability and larger surgical volume of the surgeon tended to be associated with lower odds of receiving OS.

## Discussion

We found informal networks of physicians who frequently shared patients with each other. Surgeons who interacted with peer physicians with high rates of OS were more likely to adopt OS in their own subsequent practice of postpartum and interval sterilizations. Other clinical and nonclinical factors (eg, location of care and surgeon volume) were also associated with OS adoption.

Unlike prior research studying individual or institutional factors promoting or impeding OS uptake, we applied a novel network analysis to examine the influence of relationships among physicians.^17^ Our findings suggest potential physician peer influence in OS uptake. This is consistent with the growing evidence that interpersonal connections between physicians can affect clinical practice and the diffusion of medical innovations.^31,32^ Physicians who were connected to early adopters of new treatment modalities, advanced imaging tests, and new medications were more likely to adopt these practices for their own patients.^30,33,34^ Our study extends this literature by suggesting similar associations in OS practice in the context of physician networks derived from shared patients. These networks were formed as a result of the diverse and varied paths that patients take across our fragmented health care system. Our findings reinforce the importance of studying these informal networks to better understand diffusion of practice patterns.

The ovarian cancer prevention campaign in Canada revealed that gynecologists practice in environments with varying levels of social cohesion, exhibiting different rates of OS uptake.^35^ Highly cohesive regions had respected physician champions and physicians were heavily influenced by peer pressure and did not want to be practice outliers.^35^ Less cohesive regions lacked physician champions, but many gynecologists still sought advice from peers through personal or professional connections and waited until their peers changed practices.^35^ These observations are consistent with our findings. Future efforts leveraging peer influence among physicians (eg, physician champions, shared training) may help improve OS uptake.^31,36^ The lack of a statistically significant interaction between network density and peer influence is likely because of the limited variability in network density in our sample, which warrants further assessment in future research to inform whether more interconnected networks are more effective in disseminating practice patterns.

In addition to clinical factors, we also observed nonclinical factors associated with OS adoption. For instance, patients in the South or in areas with the most vulnerable SDI were less likely to receive OS. This is consistent with prior research^5,28^ and suggests unwarranted variation in practice or access to this cancer prevention opportunity. Given that lower socioeconomic status has been associated with a higher risk of ovarian cancer diagnosis at a more advanced stage,^37^ targeted interventions to promote OS uptake in this population can be especially beneficial and warrant particular attention. Surprisingly, we found surgeons with a larger volume of sterilization procedures were less likely to perform OS. It is possible that since our sample focused on patients at low to average risk for ovarian cancer, larger-volume physicians in our study mostly cared for low-risk patients and were less concerned about cancer prevention. It is also likely that concerns about prolonged operative time associated with OS^6,7,11,14^ particularly influence higher-volume surgeons. More research on how surgical volume affects physicians’ OS uptake is needed.

### Limitations

Reliance on administrative codes was a major limitation of our study. Because different administrative codes were available in inpatient vs outpatient claims, our postpartum sterilization sample measured complete salpingectomy, but our interval sterilization sample measured any salpingectomy (complete or partial). This could confound our observed higher rate of OS among interval compared with postpartum sterilizations. Use of administrative codes may also limit measurement accuracy of other clinical conditions or procedures. Additional limitations include our lack of information on patients’ preference and physician or facility characteristics, as well as use of data from 1 single insurer which limited generalizability of our sample and our ability to assess the role of health insurance in influencing OS uptake. We also lacked information on patient race and ethnicity which has been shown to affect OS uptake with minoritized individuals less likely than White patients to receive OS.^38^ Additionally, we identified physician connections based on patient-sharing relationships in insurance claims. Although this approach has been shown to reflect actual relationships among physicians,^39^ it may not capture all interactions among physicians. Moreover, the algorithm we used to optimize groupings of physicians placed them into nonoverlapping networks. We recognize that in clinical practice, physician networks may not always be mutually exclusive.

## Conclusions

In this retrospective cohort study of patients undergoing postpartum and interval sterilizations, we observed physician peer influence in adopting OS for sterilization. This may be an underrecognized mechanism that can be leveraged in future dissemination of OS for ovarian cancer prevention. Variation in OS uptake for sterilization by nonclinical factors underscores a need to address inequity in accessing this preventive strategy.

## Notes

Supplement 1. **eFigure.** Sample Selection Flow Diagram **eTable. ***International Classification of Diseases* (ICD), *Current Procedural Terminology* (CPT), Healthcare Common Procedure Coding System (HCPCS), and Diagnosis Related Group (DRG) Codes Used in Defining Sample Eligibility Criteria and Variables

Supplement 2.
Data Sharing Statement
