# Error in Conflict of Interest Disclosures

**DOI:** 10.1001/jamanetworkopen.2025.45979

**Published:** 2025-11-06

In the Original Investigation titled “Physician Peer Influence on Salpingectomy Uptake for Tubal Sterilization and Ovarian Cancer Prevention,”^1^ published September 22, 2025, there was an error in the Conflict of Interest Disclosures. The text should have indicated that Dr Pollack serves as a consultant to Columbia University. This article has been corrected.^1^
