# Supplementary material for: Physician Peer Influence on Salpingectomy Uptake for Tubal Sterilization and Ovarian Cancer Prevention
Source: JAMA Netw Open. 2025 Sep 22;8(9):e2532998. doi: 10.1001/jamanetworkopen.2025.32998 (PMC12455378; doi:10.1001/jamanetworkopen.2025.32998)
Supplement: Supplement 1. — eFigure. Sample Selection Flow Diagram eTable. International Classification of Diseases (ICD), Current Procedural Terminology (CPT), Healthcare Common Procedure Coding System (HCPCS), and Diagnosis Related Group (DRG) Codes Used in Defining Sample Eligibility Criteria and Variables [file jamanetwopen-e2532998-s001.pdf]

Supplemental Online Content

Xu X, Long JB, Pollack CE, et al. Physician peer influence on salpingectomy uptake for tubal sterilization and ovarian cancer prevention. *JAMA Netw Open*. 2025;8(9):e2532998. doi:10.1001/jamanetworkopen.2025.32998

eFigure. Sample Selection Flow Diagram
eTable. *International Classification of Diseases* (ICD), *Current Procedural Terminology* (CPT), Healthcare Common Procedure Coding System (HCPCS), and Diagnosis Related Group (DRG) Codes Used in Defining Sample Eligibility Criteria and Variables

This supplemental material has been provided by the authors to give readers additional information about their work.

© 2025 Xu X et al. *JAMA Network Open*.

eFigure. Sample Selection Flow Diagram

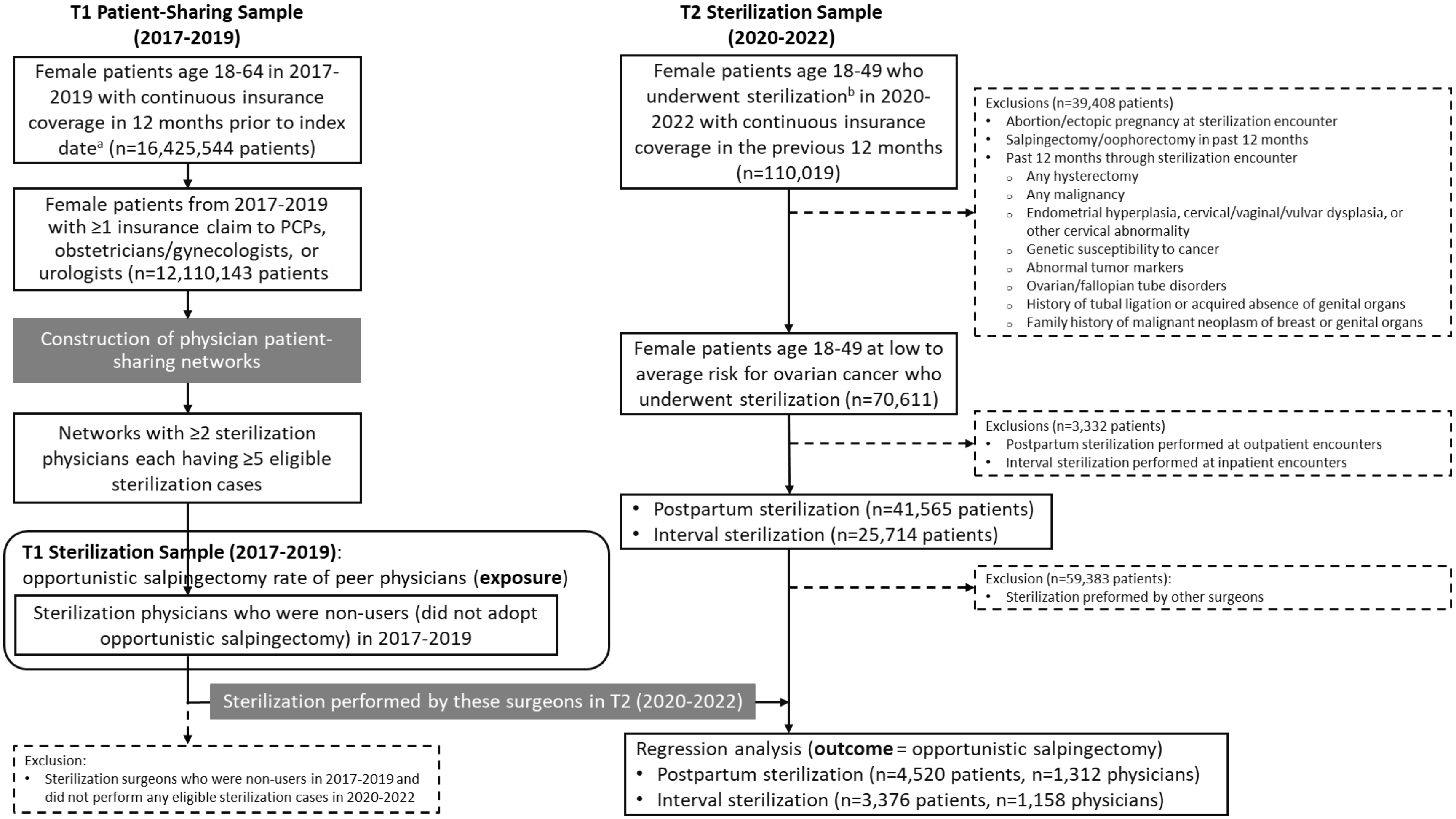

Abbreviations: PCP, primary care physician; T, time.

[a] Index date refers to the date of sterilization (for individuals who underwent sterilization) or a randomly assigned date (for individuals without sterilization)

[b] Sterilization was identified based on relevant procedure codes in conjunction with a diagnosis code for encounter of sterilization.

© 2025 Xu X et al. *JAMA Network Open*.

eTable 1. International Classification of Diseases (ICD), Current Procedural Terminology (CPT), Healthcare Common Procedure Coding System (HCPCS), and Diagnosis Related Group (DRG) Codes Used in Defining Sample Eligibility Criteria and Variables

Measure	ICD-10 Diagnosis	ICD-10 Procedure	CPT/HCPCS	DRG
Tubal sterilization[a]	Z30.2	0U550ZZ, 0U553ZZ, 0U554ZZ, 0U557ZZ, 0U558ZZ, 0U560ZZ, 0U563ZZ, 0U564ZZ, 0U567ZZ, 0U568ZZ, 0U570ZZ, 0U573ZZ, 0U574ZZ, 0U577ZZ, 0U578ZZ, 0UB50ZZ, 0UB53ZZ, 0UB54ZZ, 0UB57ZZ, 0UB58ZZ, 0UB60ZZ, 0UB63ZZ, 0UB64ZZ, 0UB67ZZ, 0UB68ZZ, 0UB70ZZ, 0UB73ZZ, 0UB74ZZ, 0UB77ZZ, 0UB78ZZ, 0UL50CZ, 0UL50DZ, 0UL50ZZ, 0UL53CZ, 0UL53DZ, 0UL53ZZ, 0UL54CZ, 0UL54DZ, 0UL54ZZ, 0UL57DZ, 0UL57ZZ, 0UL58DZ, 0UL58ZZ, 0UL60CZ, 0UL60DZ, 0UL60ZZ, 0UL63CZ, 0UL63DZ, 0UL63ZZ, 0UL64CZ, 0UL64DZ, 0UL64ZZ, 0UL67DZ, 0UL67ZZ, 0UL68DZ, 0UL68ZZ, 0UL70CZ, 0UL70DZ, 0UL70ZZ, 0UL73CZ, 0UL73DZ, 0UL73ZZ, 0UL74CZ, 0UL74DZ, 0UL74ZZ, 0UL77DZ, 0UL77ZZ, 0UL78DZ, 0UL78ZZ, 0UT50ZZ, 0UT54ZZ, 0UT57ZZ, 0UT58ZZ, 0UT5FZZ, 0UT60ZZ, 0UT64ZZ, 0UT67ZZ, 0UT68ZZ, 0UT6FZZ, 0UT70ZZ, 0UT74ZZ, 0UT77ZZ, 0UT78ZZ, 0UT7FZZ	58565, 58600, 58605, 58611, 58615, 58670, 58671, 58700, 58661	
Childbirth[b]	Z37.*, Z39.0	10D00Z0, 10D00Z1, 10D00Z2, 10D07Z3, 10D07Z4, 10D07Z5, 10D07Z6, 10D07Z7, 10D07Z8, 10E0XZZ, 0W8NXZZ, 10900ZC, 10903ZC, 10904ZC, 10907ZC, 10908ZC, 10S07ZZ, 10D17Z9, 10D18Z9	59400, 59409, 59410, 59414, 59510, 59514, 59515, 59610, 59612, 59614, 59618, 59620, 59622, 01960, 01961, 01962, 01963, 01967, 01968, 58611, 59300, 99464	Discharges before 10/1/2018: 765, 766, 767, 774, 775, 768, Discharges on/after 10/1/2018: 783, 784, 785, 786, 787, 788, 796, 797, 798, 805, 806, 807, 768
Pregnancy	O00.*-O9A.*, , Z32.*, Z33.*, Z34.*, Z36.*, Z37.*, Z39.*, Z3A.*, Z64.1*	1*	59000-59999	Discharges before 10/1/2018: 765, 766, 767, 768, 769, 770, 774, 775, 776, 777,

© 2025 Xu X et al. *JAMA Network Open*.

				778, 779, 780, 781, 782<br>Discharges on/after 10/1/2018: 768, 769, 770, 776, 779, 783, 784, 785, 786, 787, 788, 796, 797, 798, 805, 806, 807, 817, 818, 819, 831, 832, 833
Opportunistic salpingectomy[c]		0UT5*, 0UT6*, 0UT7*	58661,[d] 58700	
Oophorectomy		0UT0*, 0UT1*, 0UT2*	58720, 58940	
Exclusion (sterilization encounter)				
Procedure not carried out	Z53.0, Z53.1, Z53.2, Z53.8, Z53.9			
Abortion or ectopic pregnancy	O00.*-O08.*	10A*, 10T2*	59100, 59120, 59121, 59130, 59135, 59136, 59140, 59150, 59151, 59812, 59820, 59821, 59830, 59840, 59841, 59850, 59851, 59852, 59855, 59856, 59857, 59870, S0190, S0199, S2260, S2265, S2266, S2267	770, 777, 779
Exclusion (past 12 months through sterilization encounter)				
Malignancy	C00.*-C80.*, C7A.*, C7B.*, C81.*-C96.*, D00.*-D09.*, D37.*-D48 .*, D49.*, J91.0, R18.0, Z85.*, Z51.0, Z51.1*, Z51.5*, Z08.*		45126, 51597, 57531, 58200, 58210, 58240, 58285, 58548, 58575, 58943, 58950, 58951, 58953, 58954, 58956	
Elevated risk for cancer				

© 2025 Xu X et al. *JAMA Network Open*.

Endometrial hyperplasia, cervical/vaginal/vulvar dysplasia, or other cervical abnormality	N85.0*, R87.610, R87.611, R87.612, R87.613, R87.614, R87.618, R87.619, R87.620, R87.621, R87.622, R87.623, R87.624, R87.628, R87.629, R87.69, R87.7*, R87.8*, N87.*, N89.0, N89.1, N89.3, N90.0, N90.1, N90.3			
Genetic susceptibility to cancer	Z15.0*			
Abnormal tumor markers	R97.*			
Ovarian or fallopian tube disorders	D28.2, N83.*, Q50.*, N70			
History of tubal ligation or acquired absence of genital organs	Z98.51, Z90.7*			
Family history of malignant neoplasm of breast or genital organs	Z80.3, Z80.4*			
Hysterectomy		0UT9*	51925, 58150, 58152, 58180, 58200, 58210, 58240, 58260, 58262, 58263, 58267, 58270, 58275, 58280, 58285, 58290, 58291, 58292, 58293, 58294, 58541, 58542, 58543, 58544, 58548, 58550, 58552, 58553, 58554, 58570, 58571, 58572, 58573, 58575, 58951, 58953, 58954, 58956, 59135, 59525, S2078	
Exclusion (past 12 months)				

© 2025 Xu X et al. *JAMA Network Open*.

Removal of fallopian tubes or ovaries		0UT0*, 0UT1*, 0UT2*, 0UT5*, 0UT6*, 0UT7*	58700, 58940, 58943, 58720, 58950, 58952, 58661	
Covariates				
Body mass index category: 25-29 kg/m^2	E66.3, Z68.25, Z68.26, Z68.27, Z68.28, Z68.29			
Body mass index category: 30-39 kg/m^2	E66.09, E66.1, E66.8, E66.9, Z68.3			
Body mass index category: ≥40 kg/m^2	E66.01, E66.2, Z68.4			
Smoking status	F17.*, O99.33, T65.2, Z71.6, Z72.0, Z87.891			
Cesarean delivery	O75.82, O82	10D00Z0, 10D00Z1, 10D00Z2	59510, 59514, 59515, 59618, 59620, 59622	

[a.] Tubal sterilization was identified based on relevant procedure codes in conjunction with a diagnosis code for encounter of sterilization.
[b.] Additional ICD-10 diagnosis codes were used to identify childbirth encounters based on Sarayani A, Wang X, Thai TN, Albogami Y, Jeon N, Winterstein AG. Impact of the Transition from ICD-9-CM to ICD-10-CM on the Identification of Pregnancy Episodes in US Health Insurance Claims Data. Clin Epidemiol. 2020;12:1129-1138.
[c.] Opportunistic salpingectomy at the time of postpartum sterilization was identified using ICD-10 procedure codes (with additional requirement of no concurrent oophorectomy codes). Opportunistic salpingectomy for interval sterilization was identified using CPT/HCPCS codes (with additional requirement of no concurrent oophorectomy codes).
[d.] CPT code 58661 was considered as a measure of salpingectomy only when a concurrent diagnosis code for sterilization encounter was present and there was no concurrent procedure code for oophorectomy.

© 2025 Xu X et al. *JAMA Network Open*.
