# Supplementary material for: Physician Peer Influence on Salpingectomy Uptake for Tubal Sterilization and Ovarian Cancer Prevention
Source: JAMA Netw Open. 2025 Sep 22;8(9):e2532998. doi: 10.1001/jamanetworkopen.2025.32998 (PMC12455378; doi:10.1001/jamanetworkopen.2025.32998)
Supplement: Supplement 2. — Data Sharing Statement [file jamanetwopen-e2532998-s002.pdf]

Data Sharing Statement

Xu. Physician Peer Influence on Salpingectomy Uptake for Tubal Sterilization and Ovarian Cancer Prevention. *JAMA Netw Open*. Published September 22, 2025. doi:10.1001/jamanetworkopen.2025.32998

Data
Data available: No

Additional Information
Explanation for why data not available: This study used the Blue Cross Blue Shield Axis Database, which is a proprietary database acquired from a third party (Blue Cross Blue Shield Association). There are legal restrictions in the conditions of our data use agreement prohibiting us from sharing the data publicly. Investigators wishing to obtain the Blue Cross Blue Shield Axis Database would be able to access these data by contacting Blue Cross Blue Shield Association.
